# Somatic cell structural variant mutagenesis and neurologic disease

**DOI:** 10.1016/j.xgen.2023.100376

**Published:** 2023-08-09

**Authors:** James R. Lupski

**Affiliations:** 1 Departments of Molecular & Human Genetics, and of Pediatrics, Baylor College of Medicine and Texas Children’s Hospital, Houston, TX 77030, USA

## Abstract

Detection of organismal mosaic states for variant alleles faces technical and analytical challenges, as does the association of such variant alleles with susceptibility to neurologic disease. In this issue of *Cell Genomics*, Maury et al.^1^ reanalyze genotyping arrays of a schizophrenia cohort providing evidence for the contribution of somatic structural variant mutagenesis and rare variant alleles.

## Teaser

Detection of organismal mosaic states for variant alleles faces technical and analytical challenges, as does the association of such variant alleles with susceptibility to neurologic disease. In this issue of *Cell Genomics*, Maury et al. reanalyze genotyping arrays of a schizophrenia cohort providing evidence for the contribution of somatic structural variant mutagenesis and rare variant alleles.

## Main text

Mosaic mutations are now well established to underlie rare Mendelian disease.^2^ Eduardo Maury and colleagues provide evidence for a causative role of somatic mosaic copy number variant (sCNV) alleles, a type of structural variant (SV),^3^ in susceptibility to schizophrenia.^1^ These data support the hypothesis that somatic mutagenesis and rare variant alleles play a contributory role in both “Mendelizing” rare neurological disease traits and more common and complex neuropsychiatric conditions, such as schizophrenia. The study highlights the potential role of SV mutagenesis of somatic cells, i.e., postzygotic mutagenesis of cells during early organismal development, in the perturbations of biology in balance that contribute to organismal disease.

Germline copy number variants (gCNV), including heterozygous deletion CNVs at 22q11.21, 15q13.3, and 1q21.1 and duplication CNV at 16p11.2, were among the first specific type of genome mutations robustly associated with susceptibility to schizophrenia. gCNVs were found to underlie cognitive phenotypes both in population studies^4^ and in many clinical cohorts, but it is only more recently that postzygotic mutagenesis and somatic mosaicism has been established to contribute to organismal disease.^5^^,^^6^

Genome-wide detection of somatic mutation events are dependent on: (1) the cell(s) or tissue source from which the genomic DNA was isolated; (2) the perspective and resolution of the assay applied to investigate that personal genome and alleles at a specific locus, e.g., SNP or CGH arrays, exome sequencing (ES), or genome sequencing (GS); and (3) the computational tools (algorithms) applied to the analyses of the genome-wide data. For both ES and GS massively parallel DNA genome sequencing approaches, the read depth of coverage and allele fraction observed are important experimental parameters to measure inference of the organismal allelic state at a given locus.

Parental genome unification of the paternal- and the maternal-derived constituents composing the diploid genome of the postzygotic somatic cell (i.e., gene copy number = 2) is highly error prone in mammalian embryos.^7^ Studies of perizygotic mutation and the phenomenon of hypermutation have provided non-invasive insights into early embryonic somatic mutagenesis.^5^^,^^6^ Genome integrity in the earliest cell divisions during blastocyst formation can be particularly vulnerable to *de novo* mutation for both sCNVs and sSNVs. Analyses of somatic mutations in humans have revealed asymmetric cellular dynamics in early human embryos and the cell population dynamics of peripheral blood, informing on clonal dynamics of early human embryogenesis^8^^,^^9^ and demonstrating that phylogenies of human development can be inferred from somatic mutations. One hypothesis regarding this cell population bottleneck of the early developmental period in the human life cycle is a genome vulnerability in which maternal proteins from the egg cell undergo a gradual dilutional effect until the developing organismal endogenous DNA repair pathways are fully operative.

A key aspect of the approach by Maury et al. was to filter sCNVs that involve gene loci mutated in clonal blood disorders and focus on recurrent sCNVs involving genes previously established to contribute to cognitive traits. This enabled enhancement of experimental signal-to-noise ratio and better visualization of early developmental sCNVs. They observed *NRXN1* and *ABCB11* to be recurrently involved in sCNVs in their dataset and a burden of such sCNVs in susceptibility to schizophrenia. Interestingly, three different-sized sCNV alleles detected by 40× GS resulted in deletions of *NRXN1* exons 1–5. Breakpoint analyses from the available GS data implicated multiple SV mutagenesis mechanisms.

Systematic breakpoint junction studies of exon deletion and duplication variant alleles for all genes causing rare disease traits will likely be a rich data source both for unraveling the molecular basis of disease and for further understanding the mechanism of SV mutagenesis. Gene loci that are recurrently mutated^10^ may have an inherent genomic architecture, e.g., direct or inverted repeats including *Alu* or propensity to form unusual non-B-DNA conformers, making the locus more susceptible to genomic instability and DNA SV mutagenesis. This vulnerability to loss of genome integrity can potentially result from either a nick to the double helix backbone or a double-strand break (DSB), disrupting genome integrity. At a nicked locus, DNA replication through the gene locus can result in a collapsed fork and generation off a one ended, double-stranded DNA (oeDNA) molecule. The DNA DSB and oeDNA are likely to be repaired through different recombination or replication-based pathways.

The potential functional effects of the *NRXN1* sCNV were explored by generating Hi-C data on neurons differentiated from induced pluripotent stem cells (iPSCs) with the heterozygous deletion versus control non-engineered iPSC-derived neurons. These studies suggest allele-specific compromise of topologically associating domain (TAD) structural integrity in schizophrenia. Their working model proposes *de novo* looping in 5′ *NRXN1* deletion alleles that connects exon 6 to a putative regulatory element, promoting the formation of spurious pathogenic transcripts. Interestingly, such SV alleles could generate a potential gain-of-function mutation, as a hypermorphic or neomorphic allele, from copy number loss.

During the last decade, a better understanding of the molecular mechanisms of SV mutagenesis and the continuing establishment of allelic series of novel genes have contributed to some of the successes of re-analyses of extant genomic data but have also pointed out the limitations of such lower-resolution genome data to resolve variant alleles. One must first detect the SV allele and then identify the breakpoints to determine any predicted consequence to gene structure and phase to the paternal versus maternal allele. This pertains to both coding and non-coding variant alleles that may potentially contribute to perturbations in organismal biology. Some of these limitations in detecting and resolving variant alleles can be overcome by appropriate GS approaches. Family trio studies using long-read genome sequencing may enhance detection of both coding and non-coding variant alleles, enable phased genomes, identity of mutational signatures, and better resolution of SV, potentially uncovering more of both gCNV and sCNV variation contributing to disease.
